# Different Numbers of Conjunctive Stimuli Induce LTP or LTD in Mouse Cerebellar Purkinje Cell

**DOI:** 10.1007/s12311-024-01726-6

**Published:** 2024-08-03

**Authors:** Atsuro Daida, Tohru Kurotani, Kazuhiko Yamaguchi, Yuji Takahashi, Noritaka Ichinohe

**Affiliations:** 1 Department of Ultrastructural Research, National Institute of Neuroscience, National Center of Neurology and Psychiatry, 4-1-1 Ogawahigashicho, Kodaira, Tokyo 187-8551 Japan; 2 Division of Neurology, Saitama Children’s Medical Center, 1-2 Shintoshin, Chuo-Ku, Saitama-Shi, Saitama, 330-8777 Japan; 3 Department of Neurology, National Central Hospital, National Center of Neurology and Psychiatry, 4-1-1 Ogawahigashicho, Kodaira, Tokyo 187-8551 Japan

**Keywords:** Purkinje cell, LTD, LTP, Nitric oxide, PKC, Calcium imaging

## Abstract

Long-term depression (LTD) of synaptic transmission at parallel fiber (PF)-Purkinje cell (PC) synapses plays an important role in cerebellum-related motor coordination and learning. LTD is induced by the conjunction of PF stimulation and climbing fiber (CF) stimulation or somatic PC depolarization, while long-term potentiation (LTP) is induced by PF stimulation alone. Therefore, it is considered that different types of stimulation induce different types of synaptic plasticity. However, we found that a small number of conjunctive stimulations (PF + somatic depolarization of PC) induced LTP, but did not induce LTD of a small size. This LTP was not associated with changes in paired-pulse ratio, suggesting postsynaptic origin. Additionally this LTP was dependent on nitric oxide. This LTP was also induced by a smaller number of physiological conjunctive PF and CF stimuli. These results suggested that a larger number or longer period of conjunctive stimulation is required to induce LTD by overcoming LTP. Ca^2+^ transients at the PC dendritic region was measured by calcium imaging during LTD-inducing conjunctive stimulation. Peak amplitude of Ca^2+^ transients increased gradually during repetitive conjunctive stimulation. Instantaneous peak amplitude was not different between the early phase and late phase, but the average amplitude was larger in the later phase than in the early phase. These results show that LTD overcomes LTP, and increased Ca^2+^ integration or a number of stimulations is required for LTD induction.

**Supplementary Information:**

The online version contains supplementary material available at 10.1007/s12311-024-01726-6.

## Introduction

The cerebellum plays key roles in motor learning, such as the vestibulo-ocular reflex (VOR) [1], eye-blink conditioning [2], and hand-reaching with a prism eyeglass [3]. As the underlying synaptic mechanism of motor learning, the importance of long-term depression (LTD) of synaptic transmission from a parallel fiber (PF) to a Purkinje cell (PC) [4, 5] is well recognized for the following reasons: First, in various types of genetically modified animals, there is a close relationship between deficiency of LTD and the impairment of behavioral motor learning [6]. Second, a new optogenetic blocker of LTD reversibly impairs VOR adaptation [7]. Lastly, the sera of patients with immune-mediated cerebellar ataxia often contain elevated antibodies against mGluR1 or GluR-delta2, which are uniquely essential for LTD induction [8]. Taken together, LTD is considered as a core mechanism for motor learning, though this remains debatable [9].

Popular stimulation protocols for LTD induction include repetitive combination of PF stimulation and CF stimulation [10, 11] or somatic depolarization of PC [12, 13] (*e.g.* 300 stimuli at 1 Hz) in rodent cerebellum. Conversely, LTP, which can reverse LTD in the cerebellum, is typically induced by repetitive PF stimulation alone [14]. The importance of the stimulation protocol is explained by the increase of Ca^2+^ concentration in PC. Coesman et al. [15] delineated that chelation of intracellular Ca^2+^ during LTD induction elicited LTP instead. Furthermore, calcium imaging study showed that a combination of PF and CF stimulation causes a supralinear increase in Ca^2+^ signals in dendritic spines of PC, compared to those caused by PF or CF stimulation alone [16]. Under more physiological condition ([Ca^2+^]_o_ = 1.2 mM), pairing of PF-burst and single CF stimulation induced LTP, though this pairing caused LTD under conventional condition ([Ca^2+^]_o_ = 2.0 mM) [17], which also supported the hypothesis that LTD required higher Ca^2+^ than LTP.

However, the effects of stimulus number or duration on LTD induction require further investigation. Karachot, et al. [18] reported that 300 times (5 min) of conjunctive stimuli of PF and CF at 1 Hz induced the maximum LTD in rat cerebellar slices. Meanwhile, they indicated that 100 times stimuli caused an increase in PF EPSP slope (112–153%) in some cells, but other cells showed LTD, meaning that 100 times stimuli caused a mixed response of LTP and LTD. To clarify if a smaller number of the conjunctive stimulation could induce LTP in mouse cerebellar slices, we attempted to investigate the effect of the number of conjunctive stimuli on synaptic plasticity, utilizing the conjunction of PF-stimulation and somatic depolarization of PC, instead of CF stimulation. LTD was induced by 90 conjunctive stimuli at 0.5 Hz, while surprisingly, LTP was induced when we decreased the number of conjunctive stimulations to 30 stimuli. Therefore, we attempted to investigate the characteristics of this LTP, utilizing pharmacological tools and calcium imaging.

## Materials and Methods

### Animals

All experiments were approved by the Animal Research Committee of the National Center of Neurology and Psychiatry and in accordance with the National Institutes of Health Guide for the Care and Use of Laboratory Animals. All the animals were obtained from commercial sources. Numbers of animals used in this study are shown in Suppl. Table 1.

### Electrophysiological Studies

**Fig. 1:**
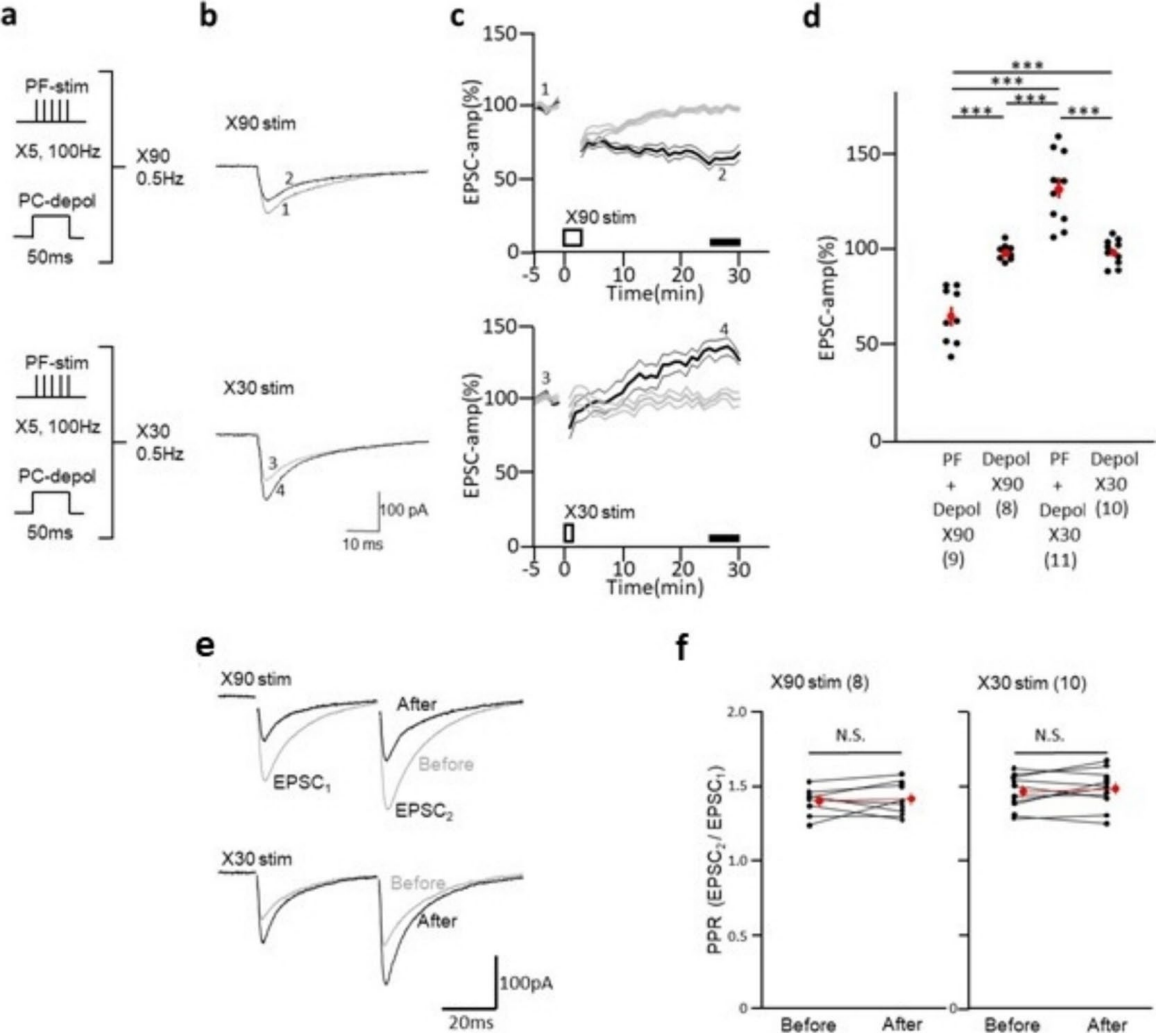
Bidirectional synaptic plasticity induced by 30 and 90 conjunctive stimulation. Schematic diagram of a conjunctive stimulation (**a**). Combinations of 5 PF stimulation at 100 Hz and 50 ms PC depolarization were applied 90 times (top) or 30 times (bottom) at 0.5 Hz. In controls, only depolarization was 30 or 90 times, following the protocol used in the previous study [13]. Representative traces of PF-EPSCs (**b**) recorded before (gray, marks 1, 3) and 26—30 min after (black, marks 2, 4) conjunctive stimulation. Five traces were averaged. Average PF–EPSC amplitude recorded from PC (**c**) before and after 90 (top panel) or 30 time (bottom panel) conjunctive stimuli (thick black line). Thin black lines indicate mean ± SEM. Thick gray lines indicate average EPSC amplitude recorded before and after PC depolarization alone. Thin gray lines indicate mean ± SEM. The bottom bar indicates the period of stimulation (open column) or comparison period (black columns, 26—30 min after stimulus onset). Scatter plot of mean amplitude of PF-EPSC (**d**) recorded 26–30 min after stimulus onset. The average of each group is represented by a red circle. Representative EPSC traces (**e**) in response to paired-pulse stimuli (50 ms apart) recorded with 90 (top panels) or 30 (bottom panels) conjunctive stimuli. Gray trace before conjunctive stimuli. Black marks after conjunctive stimuli. Scatter plots of PPR (**f**) obtained before and after conjunctive stimulation in the 90 stimulation group (left panel) or the 30 stimulation groups (right panel). Red dots represent median values with error bars. There was no significant difference in the mean values of PPR (red circles) before and after conjunctive stimulation in either group. The thin lines connect data points taken from the same cell. Bars, SEM. Numbers in parentheses indicate cell numbers. *** *P* < 0.001. Comparison between groups was performed by post hoc Tukey–Kramer test (**d**) and and paired samples t-test (**f**)

C57BL6/J male mice aged 6–7 weeks were decapitated via isoflurane inhalation to prepare cerebellar slices. The cerebellum was rapidly isolated and immersed in ice-cold ACSF containing (in mM): 125 NaCl, 2.5 KCl, 2 CaCl_2_, 1 MgSO_4_, 1.25 NaH_2_PO_4_, 26 NaHCO_3_, and 20 glucose (as described in [13]). The ACSF was bubbled with 95% (v/v) O_2_ and 5% CO_2_. The sagittal slices of the cerebellar vermis (300 µm) were prepared using a microslicer (PRO7, Dosaka) in ACSF containing 1 µM tetrodotoxin and kept in normal ACSF at 26 °C for at least 1 h. A submersion-type recording chamber was perfused with oxygenated ACSF, which contained 100 µM picrotoxin (Sigma-Aldrich) at a rate of 2 ml/min and maintained at 30.0 ± 1.0 °C. The volume of the perfusion bath was 0.5 ml. Whole-cell slice-patch recordings of the PCs were performed under an upright microscope (Eclipse E600FN, Nikon) with a 40 × water-immersion objective. Recording pipettes were pulled from borosilicate tubing and filled with a solution containing the following (in mM): For the Cs^+^-based internal solution, 60 CsCl, 40 D-gluconate, 20 TEA-Cl, 0.3 EGTA, 4 MgCl_2_, 4 Na_2_ATP, 0.4 Na_3_GTP, and 30 HEPES (pH 7.2, adjusted using CsOH). For the K^+^-based internal solution, 60 KCl, 60 K-gluconate, 0.3 EGTA, 4 MgCl_2_, 4 Na_2_ATP, 0.4 Na_3_GTP and 30 HEPES (pH 7.2). The pH of the internal solution was adjusted to 7.20 using a CsOH for Cs^+^-based solution or a KOH for K^+^-based solution. Whole-cell recordings were performed using a patch-clamp amplifier (Multiclamp 700A; Molecular Devices). The recorded signals were filtered at 3 kHz and digitized at 10 kHz. Stimulation and online data acquisition were performed using pClamp 9 software (Molecular Devices). Access resistance and input resistance were constantly monitored by applying a small hyperpolarizing voltage step (− 2 mV, 100 ms). PFs in the molecular layer were focally stimulated by applying pulses (duration, 0.1 ms) to a slice through a glass pipette (tip diameter, 5–10 µm) positioned on the surface of a cerebellar slice. CF in the granule cell layer were focally stimulated by applying pulses (duration, 0.1 ms) to a slice through a glass pipette (tip diameter, 5–10 µm) located near the Purkinje cell. The membrane potential was held at –70 mV, and a PF-EPSC was evoked at a frequency of 0.05 Hz as a test response. Under voltage-clamp conditions, LTD was induced by combining five PF stimuli and somatic depolarization (Fig. 1). For LTD-inducing conjunctive stimulation, five PF stimuli were applied at 100 Hz concomitant with somatic depolarization (− 70 mV to 0 mV, 50 ms duration) given 90 times at 0.5 Hz (3 min) [12, 13]. Under current clamp condition, a single PF stimulus combination with a single CF stimulus were repeatedly applied at 1 Hz for 1 min (Fig. 3). All the chemicals were obtained from commercial sources: Carboxy-PTIO from Dojindo (Kumamoto, Japan), and Gö6976 from Merk (Darmstadt, Germany).

### Calcium Imaging Experiment

The slices used for calcium imaging experiment were cut in the same way as electrophysiological experiments. Each slice was put on a membrane filter (Omni Pore membrane filter, JCWP01300, Millipore Corp., Billerica, MA, USA) and then transferred to an interface-type incubation chamber perfused with warm, oxygenated ACSF (30–32 °C) for recovery as previously reported [19]. After incubation for at least 1 h, we carried out the staining procedure. A calcium indicator dye (Cal-520 AM, AAT Bioquest, Inc., Pleasanton, CA, USA) was dissolved in DMSO (Sigma-Aldrich Co. LLC, St. Louis, MO, USA, dye concentration, 453 µM). The solution was aliquoted every 10 µl and stored at − 30 °C as a stock solution. The aliquoted stock solution was dissolved in 450 µl of ACSF with 5 µl of Pluronic F-127 20% solution in DMSO (AAT Bioquest, Inc., Pleasanton, CA, USA) and 5 µl of Cremophor (Nacalai tesque, Kyoto, Japan), so that the final concentration of the dye was approximately 10 µM. Each slice was transferred into a silicon well and stained with 100 µl of the staining solution for 60 min in the incubation chamber. After being well rinsed with the ACSF and left to recover for at least 30 min, each stained slice was transferred into an interface-type recording chamber (32–33 °C) and perfused with ACSF containing 100 µM picrotoxin. Neural structures in slices, such as molecular layer or PC layer, were clearly visible with a dissecting microscope. Two monopolar tungsten stimulating electrodes were placed in the PC layer and molecular layer to stimulate climbing fiber (CF) and PF, respectively (Fig. 4a).

Light emitted by a LED (LEX2, LED light source unit 40087, Brainvision Inc., Tokyo, Japan) was projected onto stained slices after being passed through an excitation filter (center wavelength, 466 nm). Fluorescence signals were conveyed through an absorption filter (transition wavelength, 506 nm) to a CCD camera (resolution: 384 × 256 pixels), and data were collected through an I/O interface (MiCAM02, Brainvision Inc., Tokyo, Japan) as a series of images at a specified sampling rate (so called “a movie”). A 1.33 × 0.88 mm^2^ imaging area was covered with a 5 × objective lens (1044723, NA: 0.5, Leica Mycrosystems, Wetzlar, Germany, Fig. 4a). In each movie, fluorescence intensities in each pixel taken from the first 8 frames were averaged and used as a reference fluorescence intensity (F) to calculate the ratio of the fluorescent transition (ΔF/F). Then, the software spatial filter (7 × 7 pixels average) with photobleaching correction was applied to each pixel in the images, and the time course of ΔF/F was determined.

First, we estimated the threshold intensities for CF or PF stimulation to induce Ca^2+^ responses in the dendritic field of PCs of the molecular layer by applying single shock to each pathway. For this purpose, a total of 256 frames (2.56 s) of ΔF/F transitions were recorded at a 10 ms sampling rate. We employed just above the threshold intensity for CF stimulation (100 to 150 µA, duration, 0.1 ms, tip-negative). PF stimulus intensity was set to no induction or if there is, a slight Ca^2+^ response with single shock, and the same stimuli applied 5 times at 10 ms interval (i.e., 5 pulses at 100 Hz) could induce a significantly larger Ca^2+^ response (30 to 50 µA, duration, 0.1 ms, tip-negative). Next, combined CF and PF stimuli with the determined intensities were applied 90 times at 2 s intervals to observe Ca^2+^ responses in the dendritic field of PCs (Fig. 4a). A total of 2730 frames of ΔF/F transitions were recorded at 10 Hz sampling rate (exposure time, 10 ms; total observation time length, 273 s).

### Statistical Analysis

Two groups were compared using Student’s *t* test, and multiple comparisons were performed via post hoc Tukey–Kramer test. A significant level of *P* < 0.05 was set.

## Results

To induce LTD of PF-PC synaptic transmission, we applied conjunctive stimulation composed of PF-burst stimuli (5 stimuli at 100 Hz) and somatic depolarization of PC (50 ms). Ninety conjunctive stimulations at 0.5 Hz for 3 min (Fig. 1a, top panel) induced LTD (Fig. 1b, c, top panels; d, 66.3 ± 4.8%, *n* = 9; 26–30 min), while PC somatic depolarization alone for 90 times did not alter EPSC amplitude (Fig. 1c, top panel; d, 99.4 ± 1.5%, *n* = 8; 26–30 min). This decrease in EPSC amplitude was significant (*P* < 0.001), and was consistent with the findings of a prior study [11]. Then we investigated the effects of a smaller number of conjunctive stimulations on PF-PC synaptic plasticity. Thirty times application of the conjunctive stimulation at 0.5 Hz induced LTP (Fig. 1a – c, bottom panels; d, 133.0 ± 5.4%, *n* = 11, *P* < 0.001) instead of small LTD, while 30 times depolarization alone did not change EPSC amplitude (Fig. 1c, bottom panel; d, 99.8 ± 2.1%, *n* = 10). Additionally, neither 30 times nor 90 times stimulation of PF burst alone induced LTP or LTD (Suppl.Fig. 1).

**Fig. 2:**
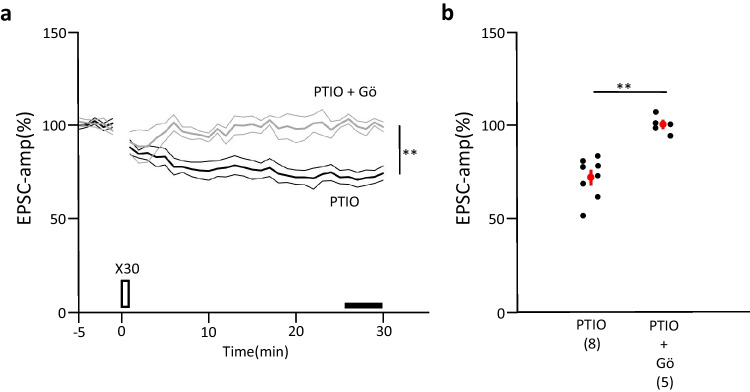
Effects of NO scavenger and PKC blocker on synaptic plasticity induced by the 30-times conjunctive stimulation protocol. Average PF-EPSC amplitude recorded before and after 30 conjunctive stimulation (thick black line) in the water-soluble NO scavenger Carboxy-PTIO (30 μM) (**a**). The thick gray line indicates the average PF-EPSC amplitude recorded with Carboxy-PTIO using a patch pipette containing Gö6976 (Gö, 0.3 μM). Thin lines indicate mean ± SEM. Scatter plot of EPSC amplitudes (**b**) recorded during 26 to 30 min after the onset of conjunctive stimulation in Carboxy-PTIO without Gö6976 (left panel) or with Gö6976 (right panel). Red circle and bars, mean ± SEM. ** *P* < 0.01. Between-group comparisons by unpaired *t*—test

Two types of LTP at PF-PC synapses are known: one type is increase in neurotransmitter release from the presynaptic terminal [20, 21], and this presynaptic type of LTP was induced by PF stimulation alone at a relatively high frequency (4–8 Hz). The other type of LTP was induced by PF stimulation alone at a relatively low frequency(1 Hz) [22], and was caused by exocytic insertion of AMPAR at the postsynaptic membrane of PC [23]. To investigate the which mechanism, presynaptic or postsynaptic, was underlying the LTP found in the present study, paired-pulse.

ratio (PPR), the relative ratio of the second EPSC amplitude to the first EPSC amplitude, was compared before and after conjunctive stimulation. Thirty conjunctive stimuli, which caused LTP, did not significantly change PPR (PPF_before_ 1.46 ± 0.04; PPF_after_ 1.49 ± 0.04, *n* = 10, *P* > 0.2) before and after the induction of LTP (Fig. 1e bottom, f right). Consistent with prior studies [15], PPR was not changed between before and after LTD induction by 90 conjunctive stimuli (1.40 ± 0.03 vs. 1.41 ± 0.04, *n* = 8, *P* > 0.7) (Fig. 1e upper, f left). Furthermore, in relation between amplitude-change and PPR, correlation (*R* = 0.454 for 30 times stimuli, *n* = 10, *P* > 0.18; *R* = 0.270 for 90 times stimuli, *n* = 8, *P* > 0.51) was not significant in each group (Suppl.Fig. 2). Also, activation of PF alone by present protocol did not induced LTP (Suppl. Figure 1). Thus, these results indirectly suggested that neither of those LTP nor LTD was caused by presynaptic changes [22, 23].

**Fig. 3:**
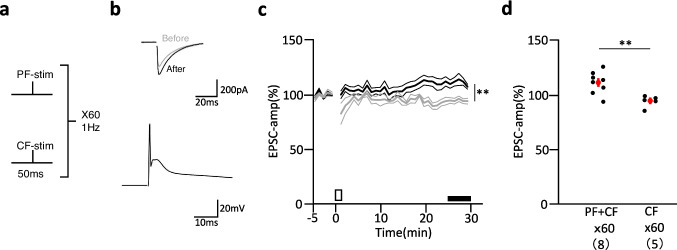
Effect of conjunctive PF and CF stimulation on PF-EPSC amplitude. Schematic diagram of a conjunctive PF and CF stimulation (**a**). Representative traces of PF-EPSCs (**b**, top panel) recorded before (gray lines) and 26–30 min after (black lines) 60 conjunctive PF and CF stimulations. Representative traces of membrane potential (**b**, bottom panel) during conjunctive PF and CF stimulation under current- clamp conditions. Holding potential was -60 to -70 mV. Average PF-EPSC amplitude (**c**) recorded before and after 60 of conjunctive stimuli (thick black line) or CF stimulation alone (thick gray line). Thin lines indicate mean ± SEM. Scatter plot of the mean amplitude of PF-EPSCs (**d**) recorded 26 – 30 min after stimulus onset. The mean for each group is represented by a red circle. Red bars, SEM. Numbers in parenthese indicate cell numbers. Comparisons between groups were performed by unpaired *t*—test. **P < 0.01

The lack of changes in PPR before and after LTP induction suggest that this type of LTP may share the mechanisms of postsynaptic LTP. Since postsynaptic LTP depends on nitric oxide (NO) [22, 23], we attempted to block mediation via NO. A water-soluble type of NO scavenger, 2-(4-carboxyphenyl)-4,4,5,5,-tetramethylimidazoline-1-oxyl-3-oxide (carboxy-PTIO, 30 μM) was added to the perfusing ACSF. LTP was entirely blocked by the NO scavenger (Fig. 3a, b), suggesting that the LTP, induced by a small number (30 times at 0.5 Hz: Fig. 1c lower) of conjunctive stimulations, was similar to postsynaptic LTP. The possibility of involvement of presynaptic LTP, which is also shown to be NO-dependent [21], seemed unlikely, since PF-burst stimulation by using the present time course (see Methods) did not cause LTP (Suppl.Fig. 1), and the PPR changes were not correlated with the change in EPSC amplitude (Suppl.Fig. 2). Importantly, under NO-free conditions, this 30-time conjunctive stimulation rather elicited a decrease in its EPSC-amplitude (Fig. 2a, b) (72.1 ± 3.7%, *n* = 8). To examine whether this decrease of EPSC shares some mechanisms with conventional LTD, which is known to depend on PKC activity [24, 25], we attempted to block PKC activity. Under NO-free conditions, the addition of a potential PKC inhibitor, Gö6976 (Gö), into the internal solution (0.3 μM) blocked LTD (100.8% ± 1.8%, *n* = 5), which indicated that this.

LTD-like phenomenon was also mediated by PKC activity. Thus, we called this LTD-like phenomenon under NO-free conditions “hidden LTD.” Together, these findings suggested that the LTP induced by 30-time conjunctive stimulation shared a common molecular mechanism with postsynaptic LTP, and hidden LTD also shared common properties with conventional LTD, at least partly.

Next, we examined whether this type of LTP was induced by physiological stimulation. We applied a conjunction of PF and CF stimulation under current clamp conditions using K^+^-based internal solution. Three hundred times conjunction of PF and CF stimulation at 1 Hz is known to induce LTD [13, 17]. When 60 times conjunction of PF and CF stimulation was applied, a small but significant increase in EPSC-amplitude (111.2 ± 3.5%, *n* = 8) was induced. This increase in EPSC amplitude was statistically significant compared to that of 60 times CF stimulation alone (94.8 ± 2.3%, *n* = 5, *P* < 0.01) (Fig. 3). Thus, physiological conjunctive stimulation consisting of PF and CF stimulation using a small number of stimuli could also induce LTP.

**Fig. 4:**
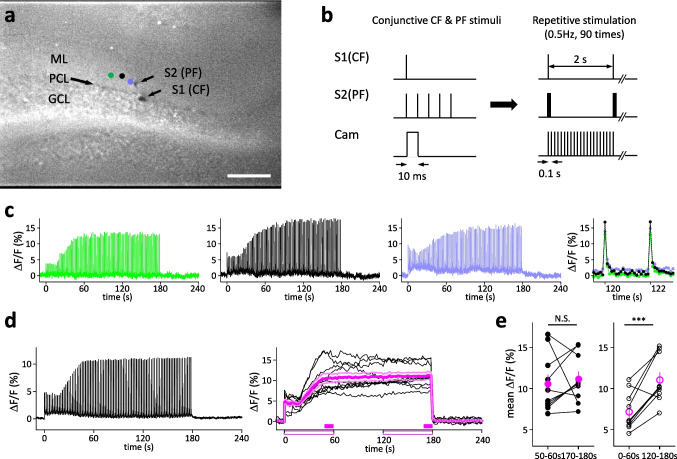
Representative example of slice image stained with Cal-520 AM Ca indicator dye (**a**). ML, PCL, GCL represent molecular layer, Purkinje cell layer (thick black arrow) and granule cell layer, respectively. S1 (CF) and S2 (PF) (indicated by two thin black arrows) are monopolar tungsten stimulating electrodes placed just below the Purkinje cell layer and molecular layer to stimulate CF and PF, respectively shows the electrode. Green, black, and purple circles indicate points where representative Ca^2+^ responses shown in (**c**) were recorded. Scale bar, 200 μm. The timing diagram of stimulation (**b**). The left panel shows the timing diagram of conjunctive CF and PF stimulation. The PF stimulation train consisted of 5 pulses (duration 0.1 ms, tip negative, applied 5 times at 100 Hz). The onset of the PF stimulation train coincided with the onset of a single CF stimulation pulse (0.1 ms duration, tip negative). The onset of image acquisition (Cam in **b**) also coincided with the onset of the CF stimulus (exposure time, 10 ms). The right panel shows that these conjunctive stimuli were applied repeatedly 90 times at 2 s intervals while the camera continuously acquired images at 0.1 s sampling intervals. Image acquisition began 20 s (200 frames) before the onset of the first stimulus and ended after acquisition of 2730 frames (i.e., movie length, 273 s). Representative example of Ca^2+^ responses (**c**) recorded from three independent points shown in (**a**). The color of each trace corresponds to the color of each circle. Time zero coincides with the onset of the first conjunctive stimulus. A time-magnified trace is shown in the right-most panel, showing that a sampling rate of 10 Hz is sufficient to track rapid changes in the Ca^2+^ response. Each dot represents the time at which the Ca^2+^ signal was sampled. The average of Ca^2+^ responses (**d**). The left panel shows the average of Ca^2+^ responses recorded from 30 points in the Purkinje cell dendritic area of 10 different slices. The right panel shows the average time course of peak Ca^2+^ responses obtained from 10 slices (black line) and the mean ± SEM of these 10 responses (thick and thin magenta lines, respectively). As in (**c**), time zero coincides with the onset of the first conjunctive stimulus. Scattered plot of changes in Ca^2+^ signal (**e**). Left panel: filled circles indicate average ΔF/F obtained from 10 different slices measured 50–60 s or 170–180 s after stimulus onset (shown as a magenta filled box in right panel of **d**). Filled magenta circles and lines are mean ± SEM and were not significantly different (*P* > 0.6, paired *t*—test). Right panel: similar to left panel, but data were acquired from 0 to 60 s and 120 to 180 s after stimulus onset (shown as a magenta open box in right panel of **d**). The average of the last 30 peak values was significantly larger than the average of the first 30 peak values (white-magenta circles and lines). Between-group comparisons were performed by paired *t*—test. ***P < 0.001

A number of reports have suggested the importance of Ca^2+^ concentration for cerebellar LTD and LTP induction [16, 23, 26]. Therefore, we conducted calcium imaging of PC dendritic region during the conjunctive repetitive stimulation. Notably, our Calcium imaging system using a CCD camera can record change in intracellular free Ca^2+^ concentration in a population of cells, but not in individual cells. Representative examples of the Ca^2+^ signals, in response to conjunctive stimulation to CF and PF applied 90 times at 0.5 Hz (Fig. 4b), are shown in Fig. 4c. These Ca^2+^ signals consisted of a sharp peak followed by baseline responses (Fig. 4c, the rightmost panel). Since the conjunctive stimulation was given at 0.5 Hz and Ca^2+^ signals were imaged at 0.1 s intervals, one peak response was observed every 20 frames. The average of the Ca^2+^ signals obtained from 30 observation points from 10 different slices indicated that the peak signal intensities gradually emerged in the dendritic region of PCs and reached their maximum values at the end of the repetitive stimuli, while the baseline signal intensities showed an initial increase followed by a gradual decrease during the repetitive stimuli (Fig. 4d, left panel). The time courses of Ca^2+^ peak responses taken from 10 slices are indicated in the right panel of Fig. 4d (right panel, thick and thin magenta lines denote the mean ± SEM). To compare the Ca^2+^ concentration at the time point of 30- and 60-times stimulation, the latest 5 or 30 stimulations (26th to 30th vs. 86th to 90th or 1st to 30th vs. 61st to 90th, respectively), were selected. The averages of peak ΔF/F observed in the 26th to 30th and the 86th to 90th stimuli (solid magenta bars, bottom of Fig. 4d, right panel) had no difference (10.6 ± 1.11 and 11.2 ± 0.899%, respectively, *n* = 10, *P* > 0.6, paired *t* test, Fig. 4e, left panel). On the other hand, the average of the peak responses observed in response to the first to 30th stimulus was significantly smaller than that observed in response to the 61st to 90th stimulus (open magenta bars, bottom of Fig. 4d, right panel) (7.23 ± 0.69 and 11.0 ± 0.89%, respectively; *n* = 10, *P* < 0.001, paired *t* test, Fig. 4e, right panel).

**Fig. 5:**
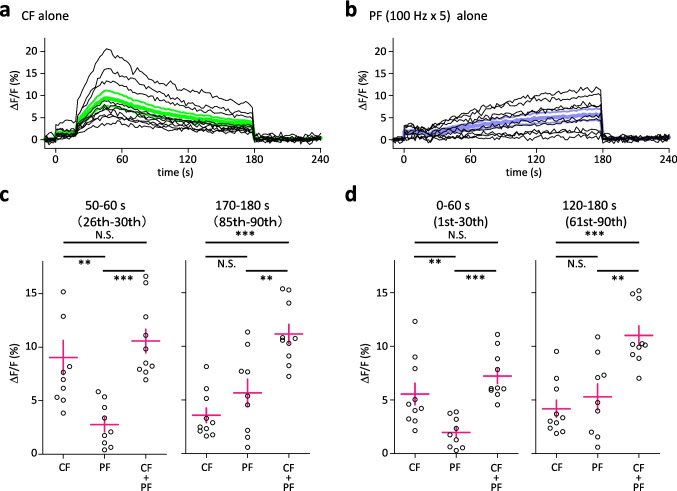
Time course of Ca^2+^ responses elicited by repetitive (90 times at 0.5 Hz) CF stimulation alone (**a**) or PF burst alone (**b**). Each black line indicates the average of 3 different observation points in the Purkinje cell dendritic area in 10 (CF stimulation) and 9 (PF burst) different slices. Colored thick and thin lines in (**a**) and (**b**) show overall mean ± SEM (*n* = 10 and 9, respectively). Scatter plot of average ΔF/F (**c**) obtained from 10 (CF alone and CF + PF) and 9 (PF alone) different slices measured 50–60 s (left panel) or 170–180 s (right panel) after the onset of CF stimulation, PF stimulation, or conjunctive CF and PF stimulation. Scatter plot of average ΔF/F (**d**) obtained from 10 (CF alone and CF + PF) and 9 (PF alone) different slices measured from 0 to 60 s (left panel) or 120 to 180 s (right panel) after the onset of CF stimulation, PF stimulation, or combined CF and PF stimulation. Each magenta cross shows mean ± SEM. Comparisons between groups were performed by post hoc Tukey–Kramer test. **P < 0.01, ***P < 0.001

Finally, we investigated the Ca^2+^ signals during PF or CF stimulation alone, which did not induce either LTP or LTD. The patch clamp experiment for stimulation of PF alone and CF alone showed no changes in EPSC (CF stimulation: Fig. 1 control experiment. PF stimulation: Suppl.Fig. 1). CF stimulation alone had an early peak of response, which was around 45 to 50 s, while PF alone stimulation showed a gradual increase, which peaked at the end of the stimulation (Fig. 5a, b). The averages of peak ΔF/F between the 26th and 30th stimuli were significantly higher for the CF + PF stimulation group (10.6 ± 1.1%, *n* = 10, *P* < 0.001) and CF stimulation alone group (9.0 ± 1.6%, *n* = 10, *P* < 0.01) than for the PF stimulation alone group (2.8 ± 0.7%, *n* = 9), while the averages of peak ΔF/F between the 86th and 90th stimuli for the CF + PF group (11.2 ± 0.9%, *n* = 10) were significantly higher than those for the CF alone group (3.6 ± 0.7%, *n* = 10, *P* < 0.001) and PF alone group (5.7 ± 1.3%, *n* = 9, *P* < 0.01). For the average of the peak responses during the longer time period (for 60 s), average peak Ca^2+^ signals were smaller in the PF-stimulation alone group (1st to 30th: 2.0 ± 0.5%, *n* = 9; 60th to 90th: 5.7 ± 1.3%, *n* = 9) than in the PF + CF stimulation group (1st to 30th: 7.2 ± 0.7%, *n* = 10, *P* < 0.001; 61st to 90th: 11.0 ± 0.9%, *n* = 10, *P* < 0.01), while average peak Ca^2+^ of the CF-stimulation alone group (4.2 ± 0.8%, *n* = 10, *P* < 0.001) was smaller than that of the PF + CF stimulation group (11.0 ± 0.9%, *n* = 10) only between the 61st and 90th response.

## Discussion

The present results showed that LTP was induced by a small number of conjunctive PF stimuli and somatic depolarization (30 stimuli at 0.5 Hz), while a larger number of conjunctive stimulations (90 stimuli) induced conventional LTD in mouse cerebellar slice [12]. This LTP had some similarities to postsynaptic LTP for the following reasons: first, PPR was not changed before and after LTP induction. Second, there was no significant correlation between amplitude change in EPSC and PPR upon LTP. Third, a carboxy-PTIO, a NO scavenger, blocked the induction of LTP. NO is known to enhance exocytic insertion of AMPA-R in the postsynaptic membrane, which is suggested to be the underlying mechanism for postsynaptic LTP [23, 27]. Presynaptic type of LTP, caused by repetitive stimulation of the PF alone at 4 Hz, was also reported to be NO-dependent [21], but stimulation of PF alone by our protocol (5 stimuli at 100 Hz were given at 0.5 Hz 30times or 90 times) did not induce LTP (Suppl.Fig. 1), so contribution of presynaptic component for LTP caused by 30times conjunctive stimulation seems unlikely in the present study.

Moreover, these findings were preserved even under physiological conjunctive stimulations, specifically conjunction of PF and CF stimulation. Because intracellular Ca^2+^ concentration is considered to be crucial for thresholding LTP and LTD, we performed calcium imaging in PC dendritic region during repetitive conjunctive stimulation. Peak values of Ca^2+^ signals measured 50–60 s and that measured 170–180 s were not different, but average Ca^2+^ signals during 0–60 s were smaller than those measured during 120–180 s (see Fig. 4). These characteristics of time-dependent change in Ca^2+^ signals suggested that the time period of the increase in Ca^2+^ signals or the numbers of stimulation is important for LTD induction. This phenomenon was consistent with a hypothesis that LTD induction requires higher Ca^2+^ concentration than that required for LTP in cerebellar PC, namely, the inverse BMC theory [15, 28, 29] and the leaky integrator theory by Tanaka et al. [30], suggesting that integrated amounts of Ca^2+^ signals are important for LTD induction.

Our experiment first showed a peculiar LTP, which was induced by smaller number (30 times) of conjunctive stimulation of PF and CF or PC somatic depolarization. In prior studies, PF stimulation alone elicit LTP [14, 15, 22, 28, 29], but under more physiological condition using 1.2 mM of [Ca^2+^]_o_, paring of PF burst stimuli and CF stimulus elicited LTP [17]. The present LTP, elicited by conjunctive stimulation of PF and CF or PC somatic depolarization, might be relevant to the report by Karachot et al. [18] who described an occasional increase in EPSP when the stimulation number of 1 Hz PF and CF stimulation protocol was decreased from 300 to 100 times. Furthermore, this LTP had no changes in PPR and was blocked by NO scavenger, suggesting that it shares some common features of postsynaptic LTP [22]. Additionally, under NO-free conditions, we found that 30-time conjunctive stimulation induced a decrease in EPSC amplitude, and this decrease was blocked by adding a potential PKC inhibitor. Since PKC is essential for LTD induction, presumably this finding was similar to the LTD reported in cultured PC under NO-free conditions [31]. Together, it is suggested that during the small number of conjunctive stimulations, the pathway of LTD and LTP was activated, but the LTP was stronger, and thus LTP was consequently observed [32].

The BCM rule is known in cortical or hippocampal LTP or LTD, which is induced by a larger or smaller increase in intracellular Ca^2+^ concentrations, respectively [33]. In the cerebellum, the inverse BCM theory is recognized, wherein higher and lower Ca^2+^ concentrations induce LTD and LTP, respectively, which is supported by patch-clamp measurements and modeling. Coesman et al. [15] presented that chelation of intracellular Ca^2+^ during the usual LTD induction stimulus (PF + CF stimulation) elicited LTP instead. In our calcium imaging experiment, average peak ΔF/F observed in a longer range (1 min, latest 30 stimulations) was significantly higher in the 61th-90th stimuli than in the 1st-30th stimuli; however, a short range (10 s, latest 5 stimulations) showed no difference (Fig. 4e). Therefore, we speculate that the peak value of Ca^2+^ itself is not enough to induce LTD, even its Ca^2+^ level is high. Similarly, PF activation at 100 Hz induced LTP, and this high-frequency activation elicited larger spine calcium transients than low-frequency conjunctive activation of PF and CF, which elicited LTD. Again, it seems to be not absolute calcium amplitudes that determine whether LTD or LTP [28]. In the present study, the duration or the count of increase in Ca^2+^ may possibly be important to induce LTD. This may be consistent with a finding that the integration of an increase in [Ca^2+^]_in_ is important for LTD induction [30]. The present results suggest that integrated calcium required for LTD is larger than that for LTP. It is unclear whether the threshold for the integrated calcium required for LTD depends on stimulating frequency. Phosphorylation of CaMKII may be a key reaction for disinhibition of the positive feedback loop that accelerates LTD induction, but the detailed mechanism of this integration is still elusive and further investigations are necessary.

The measurement of Ca^2+^ signals in the dendritic region of PC elicited by stimulation of PC alone or CF alone provided further insights. Firstly, we could confirm that conjunction of PF stimulation and CF stimulation induces the largest increase in Ca^2+^, which was consistent with the increase in [Ca^2+^]_in_ in the dendritic spine of PC [16]. Secondly, PF alone stimulation showed the weakest increase of Ca^2+^ signal in the bulk dendritic region, which could not induce even LTP (Suppl.Fig. 1). Thirdly, the peak ΔF/F of Ca^2+^ signal elicited by CF stimulation alone was comparable with that elicited by the conjunctive stimulation at the time point of 26th-30th stimulation (Fig. 5c). However, it did result in neither LTP nor LTD. These may be explicable by the following reasons. CF EPSPs activate voltage-gated calcium channels located in the dendritic branchlet [34, 35], so an increase in Ca^2+^ signal in the dendritic spine must be lower than that in the bulk dendrite region; hence, the Ca^2+^ concentration may not reach the threshold of LTD. As for the induction of LTP, mediation by NO is essential, and NO production required activation of mGluR1 and the downstream signal cascades [36–40]. Because mGluR1 is localized at PF-PC synapses [41], simple CF stimulation cannot activate the signal pathway from mGluR to NO production. Though the highest values of Ca^2+^ signals were similar between conjunctive stimulation group and CF stimulation alone group, Ca^2+^ signal increased transiently with a peak at around the 24th stimulation in CF stimulation alone group, while in conjunctive stimulation group, Ca^2+^ signal amplitude stayed at high level from the 25th to 90th　response. This difference in time course of Ca^2+^ signal suggests the importance of the duration or integrated amount of increase in Ca^2+^, which may be relevant to Ca^2+^ integration [30]. However, we need to acknowledge that detailed analysis, such as measurement of dendritic spine may add more information, indicating the need for further investigation.

The physiological role of postsynaptic LTP in PF-PC synapses is considered the reversal of LTD [14, 15]. If we assume learning without reversal of LTD, the density of AMPARs at the PF-PC synapse would markedly and persistently decrease after acquisition of various types of learning. Therefore, when PFs are activated without error signal, which is conveyed via CF [42], synaptic strength from PF to PC would be enhanced through LTP. The point of our finding is that the smaller number of conjunctive PF + CF stimulations, which include error signal, could also induce LTP. The intricate neuronal circuit of the cerebellum is thought to encode an internal model that reproduces the dynamic properties of body parts [43]. When an existing internal model for a particular performance becomes inadequate, for example, by a change in environment, error signals are generated, and the internal model would be rewritten using LTD. However, LTD may be induced accidentally by noisy perturbation; in such a case, inadequate LTD should be reversed by LTP and maintain the existing internal model. The smaller number of conjunctive PF + CF stimulations may be treated as a kind of noisy perturbation. When real alternation of the internal model is required, larger number of inputs with error signal would be able to overcome LTP and induce LTD. These findings shed light on the importance of both LTP and LTD in maintaining the internal model and learning in the cerebellum.

## Conclusion

We found that LTP, but not a small LTD, was induced by a small number of conjunctive stimuli consisting of PF stimulation and PC somatic depolarization or PF and CF stimulations. Ca^2+^ measurements showed that the instantaneous peak size of the Ca^2+^ signal did not differ between groups with fewer and more stimuli, but the 1-min averaged Ca^2+^ signal was larger in the group with more stimuli. These results suggest that LTD overcomes LTP, and that more Ca^2+^ integration or a greater number of stimuli is required to induce LTD.

## Supplementary Information

Below is the link to the electronic supplementary material.

Supplementary file1 (PDF 349 KB)

## Data Availability

No datasets were generated or analysed during the current study.
