# Supplementary material for: Different Numbers of Conjunctive Stimuli Induce LTP or LTD in Mouse Cerebellar Purkinje Cell
Source: Cerebellum. 2024 Aug 3;23(6):2297–307. doi: 10.1007/s12311-024-01726-6 (PMC11585524; doi:10.1007/s12311-024-01726-6)
Supplement: Supplementary file 1 — Supplementary file1 (PDF 349 KB) [file 12311_2024_1726_MOESM1_ESM.pdf]

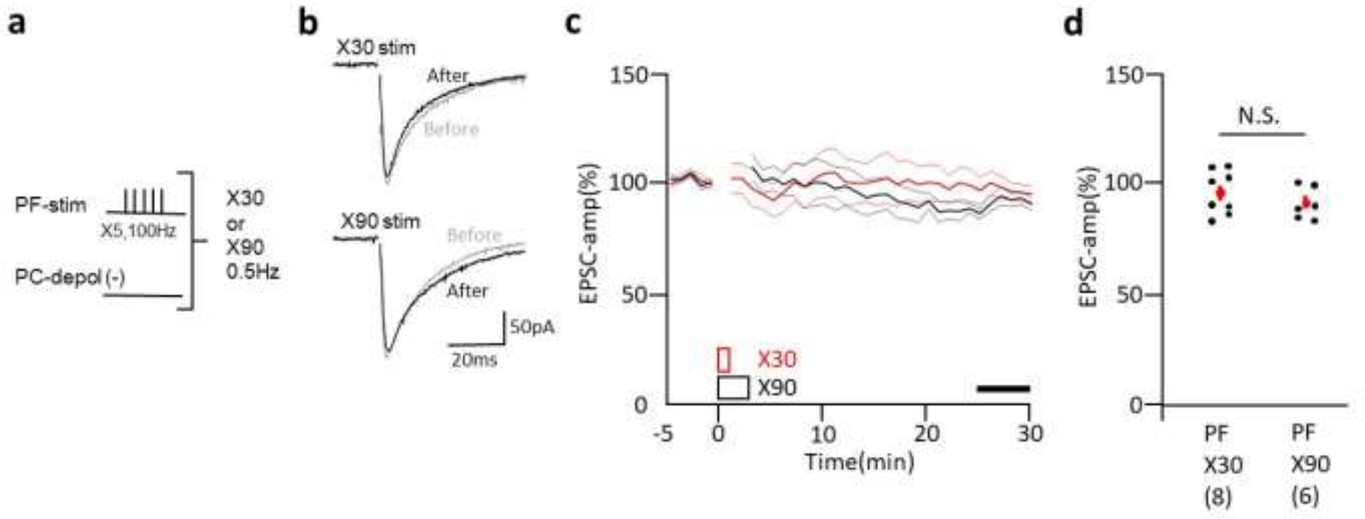

Supplementary Figure 1: Effect of PF stimulation alone on PF-EPSC amplitude. Schematic illustration of a PF stimulation alone (**a**). Five PF stimuli at 100 Hz were applied either 30 or 90 times at 0.5 Hz. Representative traces of PF-EPSCs (**b**) recorded before (gray lines) and 26-30 min after (black lines) 30 (top panel) or 90 (bottom panel) PF stimulation. Average PF-EPSC amplitude (**c**) recorded before and after 30 (thick red lines) or 90 (thick black lines) burst stimuli. Thin lines indicate mean ± SEM. Scatter plot of mean amplitude of PF-EPSCs (**d**) recorded 26 – 30 min after PF-stimulation onset. The mean for each group is represented by a red circle. Bars, SEM. Numbers in parentheses indicate cell numbers. Comparisons between groups were performed by unpaired sample *t*-test. $P > 0.3$.

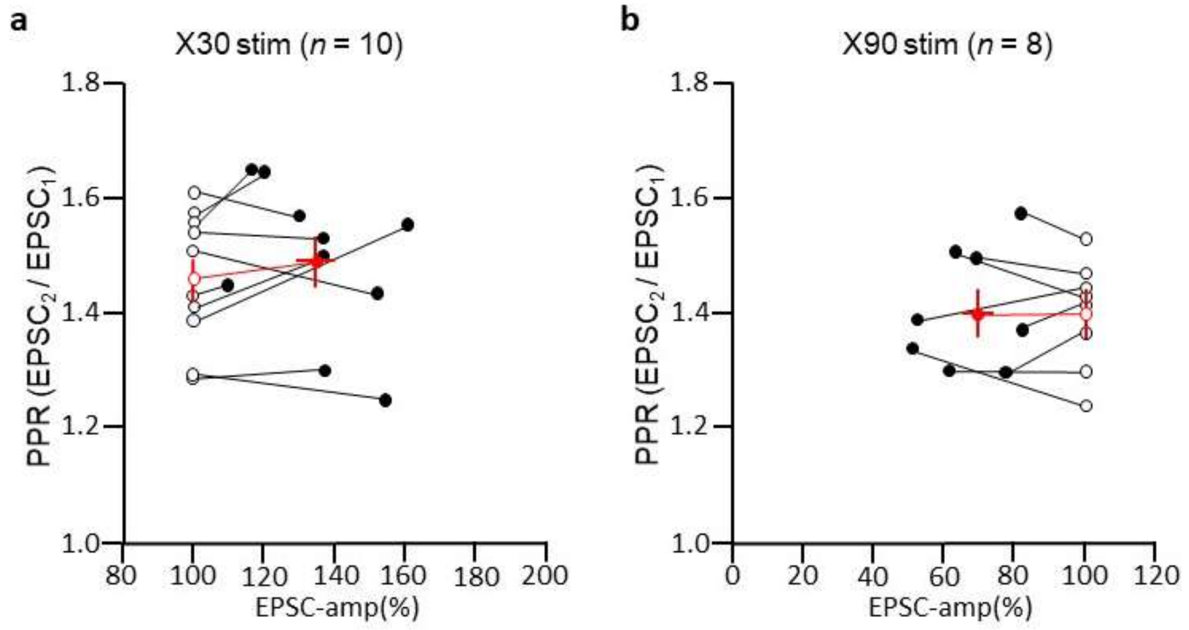

Supplementary Figure 2: Relation between EPSC-amplitude (26-30 min) and PPR in x30 (A) or x90 (B) conjunctive stimulation groups. EPSC pair for PPR-measurement was recorded just before recording of basal EPSC (empty circles) or just after 30 min from conjunctive stimulation (black circles). A. Correlation coefficient, 0.4537. $P > 0.1878$. B. Correlation coefficient, 0.2696. $P > 0.5185$.

Red circle: mean value, Red bar: SEM.

Supplementary Table 1

Table of number of cells and animals used in each experiment.

Fig.1

	Cells number	Animals number
PF+Depo x90	9	9
Depo only x90	8	8
PF+Depox30	11	11
Depo only	10	10

Fig.2

	Cells number	Animals number
PTIO	8	8
PTIO+Gö6976	6	6

Fig.3

	Cells number	Animals number
CF+PF	8	8
CF	5	5

Fig.4,5

	Slices number	Animals number
CF	10	5
PF	9	5
CF+PF	10	5

Supplementary Fig.1

	Cells number	Animals number
PF x30 stim.	8	5
PF x90 stim.	6	6

Supplementary Table 2

Parameters of statistics in each experiment

Fig. 1 d

ANOVA F = 45.806 P_F = 4.88E-12

Tukey-Kramer test

t	PF+Depo x90	Depo x90	PF+Depo x30
Depo x90	5.9750		
PF+Depo x30	11.6951	5.7586	
Depo x30	5.6861	0.06623	5.3806

Tukey-Kramer test

P_t	PF+Depo x90	Depo x90	PF+Depo x30
Depo x90	6.97E-06		
PF+Depo x30	1.60E-06	1.18E-05	
Depo x30	1.43E-05	1.00	3.31E-05

Fig.1f PPR_{before} vs. PPR_{after}

PF+Depo x90

ANOVA F=0.0528 P_F= 0.8215

Paired samples t-test t= 0.3860 P_t= 0.7111

PF+Depo x30

ANOVA F=0.2518 P_F= 0.6219

Paired samples t-test t=1.17821 P_t= 0.2689

Fig.2 PTIO vs. PTIO+Gö

ANOVA F=32.831 P_F=0.00013

Unpaired samples t-test t=5.7298 P_t=0.00013

Fig.3 PF+CF vs. CF

ANOVA F=11.3063 P_F=0.00634

Unpaired samples t-test t=3.3625 P_t=0.00634

Fig.4 e

PF+CF: 50-60sec vs. 170-180sec

ANOVA F=0.1755 P_F=0.6802

Paired samples t-test t=0.5283 P_t=0.6101

PF+CF: 0-60sec vs. 120-180sec

ANOVA F=11.2860 P_F=0.003491

Paired samples t-test t=5.2207 P_t=0.000549

Fig,5 c 50-60sec

CF vs PF vs. PF+CF

ANOVA F=11.21421 P_F=0.0003

Tukey-Kramer test

t	CF	PF
PF	3.61826	
CF+PF	0.908452	4.5025

Tukey-Kramer test

P_t	CF	PF
PF	0.0035	
CF+PF	0.6398	0.00036

Fig,5 c 170-180sec

CF vs PF vs. PF+CF

ANOVA F=17.1289 P=1.80E-05

Tukey-Kramer test

t	CF	PF
PF	1.5221	
CF+PF	5.677983	4.004366

Tukey-Kramer test

P_t	CF	PF
PF	0.2972	
CF+PF	1.80E-05	0.0013

Fig,5 d 0-60sec

CF vs PF vs. PF+CF

ANOVA F= 11.67792 P= 0.000241

Tukey-Kramer test

t	CF	PF
PF	3.2445	
CF+PF	1.550016	4.753171

Tukey-Kramer test

P_t	CF	PF
PF	0.008747	
CF+PF	0.2848	0.000186

Fig,5　d　120-180sec

CF vs PF vs. PF+CF

ANOVA　F= 14.86861　P= 4.95E-05

Tukey-Kramer test

t	CF	PF
PF	0.809162	
CF+PF	5.104897	4.15958

Tukey-Kramer test

P_t	CF	PF
PF	0.7007	
CF+PF	7.51E-05	0.000872

Supplemental Fig.1

PF only　1min vs. 3min

ANOVA　F=1.022　P_F=0.332

Unpaired samples t-test　t=1.0109　P_t=0.332

Supplemental Fig.2　EPSC-amp – PPR

x30 (1 min)

Correlation coefficient:　0.4537

t　1.4400

P　0.1878

x90 (3min)

Correlation coefficient:　0.2696

t　0.6857

P　0.5185
